# Refractory rheumatoid factor positive polyarthritis in a female adolescent already suffering from type 1 diabetes mellitus and Hashimoto’s thyroiditis successfully treated with etanercept

**DOI:** 10.1186/1824-7288-39-64

**Published:** 2013-10-14

**Authors:** Alma Nunzia Olivieri, Dario Iafusco, Antonio Mellos, Angela Zanfardino, Angela Mauro, Carmela Granato, Maria Francesca Gicchino, Francesco Prisco, Laura Perrone

**Affiliations:** 1 Department of the Woman, of the Child and of the General and Specialistic Surgery, Second University of Naples (SUN), 2, via Luigi De Crecchio, Naples 80138, Italy

**Keywords:** Type 1 diabetes mellitus, Autoimmune Hashimoto's thyroiditis, Juvenile idiopathic arthritis, Polyarthritis, Rheumatoid factor, Autoimmunity, Immunotherapy, Tumor necrosis factor, Anti-TNF therapy, Etanercept

## Abstract

Type 1 diabetes mellitus may be associated with many autoimmune diseases with the common autoimmune pathogenesis. We describe the case of a girl suffering from Type 1 diabetes mellitus and autoimmune Hashimoto's thyroiditis since the childhood and, due to the onset of Juvenile Idiopathic Arthritis during adolescence, for three years practiced therapy with an anti-TNF drug, etanercept . Currently her inflammatory markers are normal, arthritis is inactive and diabetes is well controlled. During the treatment with anti-TNF drug we observed a significative reduction of insulin dose, probably due to an increased tissue sensitivity secondary to the suppression of the activity of TNF-alpha. Several clinical trials that have evaluated the effect of immunomodulatory agents in diabetic patients, especially in those with recent onset of disease, were already performed but further studies of longer duration on a larger population are needed to assess the role of biologic drugs and immunotherapy in this group of patients.

## Background

The coexistence of Juvenile Idiopathic Arthritis (JIA) with Type 1 Diabetes Mellitus (T1DM) and Autoimmune Hashimoto's Thyroiditis (AHT) may be considered rare and it suggests a common genetic susceptibility [1,2]. The HLA, CTLA4 and PTPN22 genes, which regulate the activation of T-lymphocytes, have been associated with specific organ autoimmune diseases and some of their variants increase the risk of onset of these three diseases [1,3]. We describe the case of a female patient suffering since the childhood from T1DM and AHT and in therapy with insulin and L-tiroxine, who developed JIA during adolescence unresponsive to conventional therapy with Non Steroid Anti Inflammatory Drugs (NSAIDs) and Methotrexate (MTX) for which we started anti-TNF therapy. In our patient after three weeks from the introduction of etanercept, arthritis appeared in remission, without disrupting her metabolism. Upon treatment with etanercept, the daily insulin requirement was reduced, probably due to an increased tissue sensitivity secondary to the suppression of activity of TNF-alpha. Recently, a small randomized pilot study has reported that this medication in addition to being safe and effective, would be able in patients with T1DM of recent onset to prolong endogenous insulin production thus suggesting the preservation of beta cell pancreatic function [4]. Several clinical trials that have evaluated the effect of immunomodulatory agents in diabetic patients, especially in those with recent onset of disease, were already performed [5,6], but further studies with a longer follow-up are needed to assess the effectiveness and safety of immunotherapy in this group of patients [5-7].

## Case presentation

**Figure 1:**
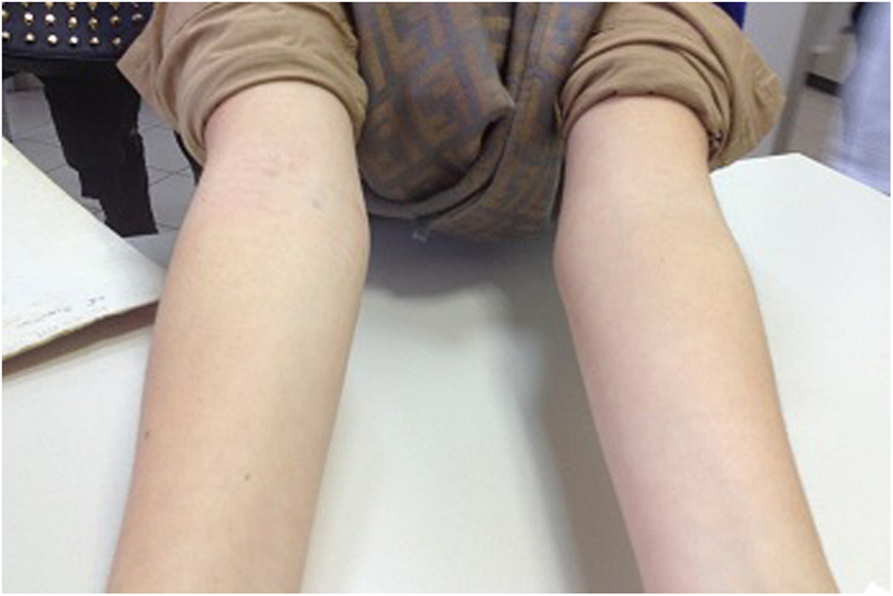
Etanercept led to a marked improvement in arthritis.

**Figure 2:**
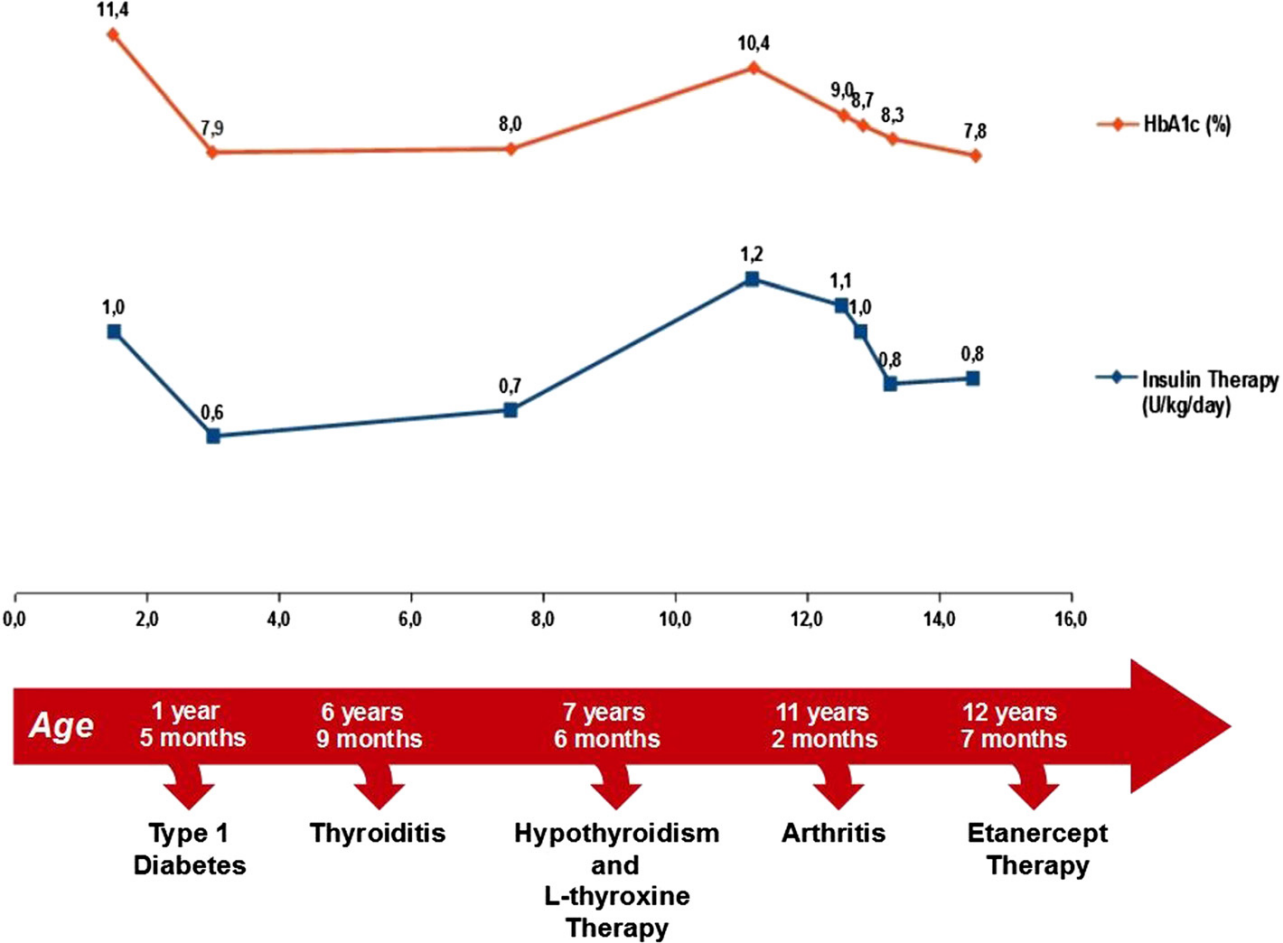
Changes over time in insulin dose and HbA1c.

The patient was in follow up in the Pediatric Diabetological Center of our Department because developed type 1 diabetes at the age of 1 year and 5 months with the signs of ketoacidosis preceded by polyuria, polydipsia, weight loss and progressive weakness. Blood tests and laboratory revealed on that occasion: pH 7.16, pCO2 35.4 mmHg, O_2_ saturation 66%, base excess - 14.8, bicarbonate 12.5 mEq/L, glucose 587 mg/dl, HbA_1c_ 11.4% (101 mmol/mol), serum C-peptide 0.2 ng/ml (n.v. 0.6 -3.7) weakly positive ICA, GADA 0.1 AU/ml (n.v. <3), IA-2 autoantibodies 33 AU/ml (n.v. <1). The family history revealed that a maternal uncle was suffering from diabetes mellitus since the age of 31 years old, treated initially with oral hypoglycemic drugs and later with insulin. AHT was diagnosed at the age of 6 years and 9 months because the presence of autoantibodies (anti-TgAbs 181,80 IU/ml and anti-TPOAbs 578.90 IU/ml) and because of the findings of the ultrasound (US) and Doppler-US that showed respectively a heterogeneous echogenicity of the thyroid with multinodular hypoechoic areas and a widespread hypervascularization. It was necessary to introduce replacement therapy with L-thyroxine 9 months later for the onset of hypothyroidism (fT3 4.18 pg/ml, fT4 6.11 pg/ml; TSH 64.41 μIU/mL). At the age of 11 years and 2 months old she was referred to the Pediatric Rheumatological Center of our Department for persistence, from approximately 6 months, of diffuse arthralgia, morning stiffness lasting about an hour and lameness. The insulin and thyroid replacement therapy had been adequate up to that moment: in fact, her reference percentiles for height (145 cm), weight (39.5 kg) and BMI (18,8 ) were all included between the 25th and the 50th percentile [8] and the path of her growth curve had always continued along this channel, without showing deflections despite the medical history revealed that the patient suffered from the joint symptoms since at least 6 months and her metabolic state was very altered (HbA1c 10.4%; insulin dose of 1.2 Units/Kg/day). Pubertal development was appropriate to sex and age (P_2_B_2_) [9]. Physical examination showed the presence of all the signs of arthritis of the elbows, wrists, hips, knees, left ankle, metacarpophalangeal joints of the third and fifth finger of the left hand and first and third finger of the right hand. Laboratory tests found inflammatory anemia (hemoglobin 8.1 g/dl, ESR 125 mm/hour, CRP 25.8 mg/dl, fibrinogen 682 mg/dl, serum iron 8 mcg/dl, ferritin 210 ng/ml) and bone marrow aspirate excluded malignancies. The serological tests for celiac and connective tissue disease (ENA) were negative, but positive for rheumatoid factor (52 umol/L) and for ANA (1:160); the eye examination excluded the presence of concomitant iridocyclitis. Considering 2 further test for rheumatoid factor (RF) resulted positive (28 umol/L, 33 umol/L; n.v. 0-20) at 3 months apart during the first 6 months of disease, the diagnosis of RF positive polyarthritis was made according with International League of Associations for Rheumatology (ILAR) classification criteria [10] and treatment with anti-inflammatory drugs (Naproxen, 15 mg/kg/day) and MTX at a dose of 15 mg/m^2^/week was assigned according to 2011 American College of Rheumatology (ACR) reccomandation [11]. After about a year of relatively good control of the arthritis, the patient had a progressive clinical deterioration: the physical examination showed limitation of flexion and extension of both wrists, elbows and the left ankle which was also swollen. In view of this clinical picture and the persistence of inflammatory anemia (9.8 g/dl, ESR 50 mm/hour, CRP 13.6 mg/dl) we decided to start anti-TNF therapy (etanercept 0.4 mg/kg, subcutaneously, twice in a week). We have decided not to suspend MTX, the effectiveness of which is sustained by good-quality studies [12], because the patient had always shown to tolerate it well in combination with conventional therapy based on NSAIDs although over time this treatment proved insufficient to control arthritis, and to associate it with the etanercept to enhance their mutual anti-inflammatory and immunomodulatory activities [13]. The functional limitation of the affected joints was progressively reduced and the current examination show only a slight functional limitation of both wrists in the absence of further clinical and biochemical signs of acute inflammation (ESR 13 mm/hour; CRP 0.1 mg/dl) (Figure 1). Also the metabolic control of diabetes improved and the HbA1c after three months from the starting of biological therapy was 8.7% (71 mmol/mol) and after 6 months was 8.3% (67 mmol/mol) while the insulin dose decreased respectively to 1.0 and 0.8 units/Kg/day (Figure 2). The patient continues to get the beneficial effects of etanercept treatment and at the moment both the daily requirement of exogenous insulin (0.8 U/kg/day), and the HbA_1c_ (7.8%, 61 mmol/mol) are significantly reduced compared to those found at the diagnosis of arthritis. The current blood levels of fasting C-peptide remains unchanged compared to those found at onset of diabetes (0.07 ng/mL). Furthermore, the daily requirement of L-thyroxine is stable (1.7 mg/kg/ day) and the arthritis is under control thanks to etanercept. Until now, laboratory tests performed during follow-up for celiac disease (ATA-IgA 2.5 U/ml; anti-gliadin peptides IgG 3.1 U/ml, anti-gliadin peptides IgA 1 U/ml), Addison's disease (cortisol 7.3 mcg /dl, ACTH 16.9 pg/ml) were normal, as well as the current thyroid profile (fT3 3.2 pg/ml, fT4 12.2 pg/ml; TSH 0,305 μUI /mL). From the clinical onset of joint symptoms to date, the patient has grown regularly along its growth channel (25th - 50th percentile) and pubertal development is continued normally. Currently, she shows no signs of nephropathy or neuropathy, does not suffer from high blood pressure and eye examination has ruled out so far the presence of diabetic retinopathy or iridocyclitis.

## Discussion

Few cases have been reported about the association of autoimmune diseases such as T1DM, HT, and JIA. Most of these patients are predominantly female, diabetes mellitus appeared first, followed by thyroiditis and, finally, by arthritis. This may be due to the fact that some genes involved in the pathogenesis and/or in susceptibility of these three lymphocyte T autoimmune diseases have a gender-dependent penetrance [1]. To date, insulin replacement therapy remains the mainstay of treatment of patients with T1DM. Over the past 30 years, the introduction of multiple insulin formulations, each of which has a different duration of action, has enriched the therapeutic armamentarium for the management of diabetes. The continuous development of improved insulin pumps and glucose monitoring systems more efficient will further improve glycemic control in these patients [6]. T1DM is a chronic and predictable autoimmune disease with identifiable progressive stages for which there could be the possibility of preventing progression to clinical symptoms [14,15]. Currently, several studies are evaluating the role of immunomodulatory agents and biological drugs for the prevention and therapy of T1DM. A therapy that prevents the destruction of the pancreatic tissue would be the ideal, because exogenous insulin can’t equal the precision of endogenous hormone secretion [6,16]. The immunotherapy aims to prevent the onset of autoimmune phenomena in subjects at risk (primary prevention) or counteract the autoimmunity before T1DM occurs (secondary prevention) or stop the cellular destruction and protect the remaining functional beta cells after the clinical diagnosis of diabetes (tertiary prevention) [5]. Once the strategies to prevent T1DM will be identified, reliable and cost-effective screening technologies will be also needed to identify people at risk [14,17]. The strategy of immunotherapy is based on the inhibition of pathogenic cells or in stimulating the pathways that suppress them. Non-antigen specific immunomodulators or inhibit T-cell proliferation or act directly on pathogenic B and T lymphocytes, co-stimulatory molecules, cytokines involved in beta-cells destruction. Islet antigens specific immunomodulators aim to reach and get a tolerance of long-term through the introduction of a self-antigen capable of modulating the cellular activity and turn off the inflammation [5]. Insulin antibodies can be detected in T1DM patients still before being treated with exogenous insulin and several preproinsulin (PPI)/insulin epitopes are recognized by T lymphocytes. In view of this ipothesis and these findings, the mucosal administration (oral or nasal) of insulin has been practiced clinically to try to restore the immunological tolerance against antigens insulin. However, the insulin mucosal immunotherapy was found to be incapable of preventing or delaying T1DM probably because the administration of insulin would be anticipated by the phenomenon of “antigen spreading”, that is the progressive involvement of other PPI epitopes or other beta cell antigens in autoimmune process. There is no a definitive evidence that PPI/insulin is the “initiating antigen” in human T1D and then further studies are needed to define the role of PPI/insulin antigenicity in different human populations. In fact, about 20 antigens have been identified as targets of diabetogenic T cells so far and the list is constantly growing. Other strategies have shown the ability to reduce the incidence of T1DM in nonobese diabetic (NOD) mice as the intramuscular injection of a plasmid DNA vaccine encoding proinsulin II and agonistic insulin mimetope, but their clinical utility must be determined [6]. Several clinical trials using antigen-specific therapies and immunomodulating agents have been performed, but most of them turned out to be or excessively toxic or unable to determine a long-term protection of the beta cells [6], supposedly because they have not taken into adequate consideration the fact that the residual beta cell mass and the autoimmunity differ from patient to patient [5]. It is necessary not only to identify suitable biomarkers of beta cell mass and the level of autoimmunity of every diabetic patient, but also efficiently define the formulation, dose, frequency and route of administration of the immunomodulating agents before designating future clinical trials. In addition, a better understanding of progression and mode of triggering the autoimmunity in patients with T1DM and further development of cell-based therapies and alternative sources of beta-cells for patients without beta-cellularity should precede the planning of clinical trials. The immunotherapy should be adapted to the condition of each patient's disease and is currently limited by the lack of efficient immunoreactive to beta cell antigens and appropriate biomarkers to assess the residual beta cell mass and correlating with a successful induction of a protective immune response following an antigen-specific therapy. The availability of such biomarkers would be particularly useful and important before exposing patients, particularly children and prediabetic individuals clinically healthy, at the reagents developed to interfere with their immune system [5]. The possible development in the future of biomarkers capable of staging the disease more accurately and to predict the rate of progression may help to stratify patients for future clinical trials [14]. In the context of immunotherapy of diabetes, TNF-alpha is a rational target not only because is cytotoxic to pancreatic beta cells [4,7], but also because some studies have found high levels of this cytokine compared to controls in subjects in whom had been recently diagnosed with T1DM [4]. These values remain high even when the function of pancreatic beta cells is completely compromised, making hypothesize that hyperglycemia is associated with the persistence of chronic inflammation [4]. Some paradoxical effects of TNF-alpha reported in the literature and those of anti-TNF-alpha described in several case reports may raise doubts about the application of immunotherapy in these patients [16]. In fact, it was described a case of T1DM began five months after the start of therapy with etanercept in a 7 year old girl suffering since the age of 4 years from polyarticular JIA. The development of diabetes in this patient could be considered independent from the drug since it is known that JIA and T1DM can coexist. Furthermore, this child had a family history of T1DM and positivity for anti-GAD autoantibodies even before starting therapy with etanercept [18]. Theoretically, biological therapy may have triggered prematurely diabetes in this patient predisposed, so it is important not to exclude the possibility that new autoimmune processes may manifest during therapy with etanercept in patients with JIA [18], as well as alterations in glycemia [19,20]. In fact, a 55 year old woman suffering from the age of 30 from T1DM and afflicted by rheumatoid arthritis 3 years after had several episodes of severe hypoglycemia following the administration of etanercept and adalimumab, probably due to an increased sensitivity to the action of insulin due to blockade of the action of TNF-alpha in adipose tissue level [19]. Several episodes of hypoglycaemia after the initiation of anti-TNF-alpha therapy have been reported in two women with rheumatoid arthritis, one also affected by type 2 diabetes [20] and the other by latent autoimmune diabetes of adults (LADA) [21], for which it was necessary to reduce, in both of them, the doses of exogenous insulin [20,21]. In an elderly man suffering from type 2 diabetes and psoriasis treated with etanercept, insulin was gradually reduced and finally replaced with rosiglitazone and repaglinide for frequent episodes of hypoglycaemia occurred [22]. The case of a woman suffering from JIA who developed T1DM during treatment with etanercept has been also described. This patient also had positivity for anti-GAD autoantibodies before starting biological therapy, which in this case was found to be ineffective in preventing the development and progression of diabetes [23]. On the hypothesis that anti-TNF therapy may preserve the function of pancreatic beta cells, etanercept was introduced into the treatment regimen of a 48 year old man at a distance of four months from the diagnosis of LADA allowing to keep a low insulin dose and good metabolic control during his eleven years of disease [24]. By the way, a small randomized, controlled, double-blind trial lasted 24 weeks has been recently realized in eighteen patients (11 males and 7 females, aged between 7.8 and 18.2 years) with T1DM recently diagnosed (since about 4 weeks) who received placebo or etanercept. The purpose of this study was to collect data on the capacity and effectiveness of etanercept to prolong endogenous insulin production and was founded on hypothesis that the administration of etanercept could extend the period of partial remission of diabetes (“*honeymoon*”). The authors noted lower glycosylated hemoglobin and increased endogenous insulin production with consequent reduction of exogenous requests in the group of patients treated with etanercept. They concluded that the administration of etanercept at doses used for the treatment of JIA is well tolerated in pediatric patients with T1DM type 1, protects the function of pancreatic beta cells by determining the reduction in insulin requirements and improving glycemic control [4]. These observations are very interesting, but deserve further and more extensive research as it is simplistic to assert that etanercept improves the function of beta-cells by reducing the levels of TNF-alpha, since this cytokine plays a much more complex in the progression of diabetes [7]. Considering the disappointing results of almost all clinical trials based on the use of only one immunomodulatory agent, a future approach in hindering the progression of diabetes could be the combined use of substances which have already been tested for T1DM or other autoimmune diseases, and which have a synergistic or complementary mechanisms of action. Etanercept is an ideal candidate for a combined therapeutic approach. Such combination therapy may include the following components: 1) an agent depleting T effector cells, ideally given when islet specific effector responses are at their peak; 2) an anti-inflammatory agent given for a short period at the induction of therapy; 3) an agent that induces antigen-specific regulatory T-cells, ideally given at the time point when the number of the T-regs specific for beta-cells antigens is declining; 4) an agent promoting beta-cell regeneration or the transplantation of beta-cells [5]. Many therapeutic approaches for the generation of new beta cells have been studied so far. Induction therapy of beta cells that have any chance of success must be accompanied by a powerful immunotherapy that protects newly formed cells, which would otherwise be destroyed by the autoimmune T lymphocytes. But the autoimmunity is not the only obstacle to the clinical application of beta cell regeneration. In fact, an efficient gene therapy that induces beta cell neoformation in vivo is still based primarily on viral vectors, which present themselves a significant immunogenicity and toxicity. Moreover, the neogenesis of beta cells mature in vitro from pluripotent stem cells is still insufficient to obtain a clinically relevant number of cells for a therapy. Finally, a further obstacle to the clinical application is given by the oncogenicity of pluripotent cells and thus preclinical studies are needed to overcome these limitations. A possible successful strategy would be to counter the autoimmunity with a strong short-term immunosuppression (FcR non-binding anti-CD3 mAb, anti-CD20 mAb or ATG) and prevent autoimmune reaction with long-term or intermittent immunotherapies that target costimulatory signals or cytokines (CTLA-4-Ig or IL-1 blocker) [6]. These combinated therapies are limited by the fact that there are actually few preclinical studies testing combinations of immunomodulatory agents and because it would be difficult to differentiate the individual contribution of efficacy and toxicity of two or more drugs when administered simultaneously [5]. A suggestive target of a future immunotherapeutic approach is offered by the trimolecular complex that is represented by HLA molecules, autoantigenic peptides and the TCR of a CD4 cell. A plausible therapeutic strategy could be offered by small molecules that block the presentation of the autoantigen or the recognition by the TCR of MHC-autoantigen complex or monoclonal antibodies against the trimolecular complex [5,25]. However, the effects that would result from blocking the entire molecule MHC II are still uncertain and risky [5,6]. A further opportunity of developing safe and effective immunotherapies in the future could result from further research whose purpose is to define and improve the knowledge on the molecular basis underlying the T cell response to pancreatic islet antigens in T1DM [6].

## Conclusions

We decided to treat our patient suffering from JIA with an anti-TNF-alpha therapy because unresponsive to conventional therapy with NSAIDs and MTX. Moreover, a recent randomized, double blind, placebo controlled trials has shown that etanercept appears to be well tolerated from children suffering from T1DM and that if administered within 4 weeks after the onset of diabetes would be able to preserve the function of pancreatic beta cells. This case report demonstrates that etanercept is really effective and safe for the treatment of JIA associated with T1DM and AHT. In fact our patient, in which diabetes was debuted long before arthritis and so its period of partial remission (*honeymoon*) was already exhausted, has not presented until now none of the side effects associated with this therapy apart from few episodes of hypoglycemia resolved with reduction in insulin requirements and probably due to increased tissue sensitivity induced by suppression of TNF-alpha activity. We do not exclude an additional beneficial effect resulting from the fact that we have followed the patient with much more attention and frequency due to her refractory arthritis and to the administration of a drug such as etanercept, whose adverse effects are not well known. For these reasons, we also asked the patient to increase the frequency of the home glucose monitoring and pay particular attention to diet. The presumed mechanism of action of etanercept, according to which it would improve the function of the beta-cells by counteracting the action of TNF may not have played a role in the case of our patient, since her diabetes was clinically began about 10 years before and serum concentrations C-peptide did not increase upon treatment with etanercept. Further and longer studies on a larger group of patients are needed to evaluate the ability of etanercept and other biological drugs in preventing or delaying the development of diabetes and to assess its safety, efficacy and tolerability in patients with JIA and T1DM. A better knowledge of the genetic mutations and cytokine pattern are essential to undertake targeted and more effective therapies in different subgroups of patients.

## Consent

Written informed consent has been obtained from the patient and her relatives for the publication of this case report and 2 accompaying figures. A copy of the written consent is available for review by the Editor in Chief of this journal.

## Abbreviations

TNF: Tumor necrosis factor; HLA: Human leukocyte antigen; CTLA4: Cytotoxic T-lymphocyte-associated protein 4; PTPN22: Protein tyrosine phosphatase non-receptor 22; HbA1c: Glycosylated hemoglobin; ICA: Islet cell autoantibodies; GADA: Glutamic acid decarboxylase autoantibodies; IA-2: Insulinoma associated antigen-2; Anti-TgAbs: Anti-thyroglobulin antibodies; Anti-TPOAbs: Anti-thyroid peroxidase antibodies; BMI: Body mass index; ESR: Erythrocyte sedimentation rate; CRP: C-reactive protein; ENA: Extractable nuclear antigens; ANA: Anti-nuclear antibody; ATA: Anti-transglutaminase antibodies; T-reg: Regulatory T; FcR: Fc receptor; CD3: Cluster of differentiation 3; CD20: Cluster of differentiation 20; mAb: Monoclonal antibody; ATG: Antithymocyte globulin; IL-1: Interleukin-1; TCR: T-cell receptor; CD4: Cluster of differentiation 4; MHC: Major Histocompatibility Complex.

## Competing interests

The authors declare that they have no competing interests.

## Authors’ contributions

FP and DI have made the diagnosis of type 1 diabetes mellitus, autoimmune thyroiditis and hypothyroidism and take care of the patient. They have participated in the conception and design of the manuscript, revisiting it. ANO formulated the diagnosis of arthritis, take care of the patient, participated in the conception and design of the manuscript, revisiting it. AM take care of the patient, formulated the diagnosis of arthritis and conceived, wrote and revisited the manuscript. AZ, AMA, CG, MFG and LP take care of the patient and have been involved in drafting the manuscript and revisiting it. All authors read and approved the final manuscript.

## Authors’ information

Alma Nunzia Olivieri, doctor and researcher in pediatrics. Pediatric rheumatological center.

Dario Iafusco, doctor and researcher in pediatrics. Pediatric diabetological center.

Antonio Mellos, medical doctor, intern in pediatrics. Pediatric rheumatological center.

Zanfardino Angela, pediatrician. Pediatric diabetological center.

Angela Mauro, medical doctor, intern in pediatrics. Pediatric rheumatological center.

Carmela Granato, medical doctor, intern in pediatrics. Pediatric rheumatological center.

Maria Francesca Gicchino, medical student. Pediatric rheumatological center.

Francesco Prisco, professor of Pediatrics. Pediatric diabetological center.

Laura Perrone, professor of Pediatrics and director of the Department.
